# Association between pediatric postoperative delirium and regional cerebral oxygen saturation: a prospective observational study

**DOI:** 10.1186/s12888-024-05832-x

**Published:** 2024-05-15

**Authors:** Kexian Liu, Nan Lin, Ting Jin, Yujun Xiang, Jiahuan Li, Dengming Lai, Hongzhen Xu

**Affiliations:** 1 Nursing Department, Children’s Hospital, Zhejiang University School of Medicine, National Clinical Research Center for Child Health, Hangzhou, Zhejiang 310052 China; 2 Department of Neonatal Surgery, Children’s Hospital, Zhejiang University School of Medicine, National Clinical Research Center for Child Health, Hangzhou, Zhejiang 310052 China; 3 Third Affiliated Hospital of Zhengzhou University, Zhengzhou, Henan Province China

**Keywords:** Regional cerebral oxygen saturation, Postoperative period, Delirium, Pediatrics

## Abstract

**Background:**

Postoperative delirium (POD) represents a prevalent and noteworthy complication in the context of pediatric surgical interventions. In recent times, a hypothesis has emerged positing that cerebral ischemia and regional cerebral oxygen desaturation might serve as potential catalysts in the pathogenesis of POD. The primary aim of this study was to methodically examine the potential relationship between POD and regional cerebral oxygen saturation (rSO_2_) and to assess the predictive and evaluative utility of rSO_2_ in the context of POD.

**Methods:**

This prospective observational study was conducted at the Children’s Hospital, Zhejiang University School of Medicine, Zhejiang, China, spanning the period from November 2020 to March 2021. The research cohort comprised children undergoing surgical procedures within this clinical setting. To measure rSO_2_ dynamics, cerebral near-infrared spectroscopy (NIRS) was used to monitor rSO_2_ levels both before and after surgery. In addition, POD was assessed in the paediatric patients according to the Diagnostic and Statistical Manual of Mental Disorders Fifth Edition (DSM-5) criteria. The analysis of the association between the rSO_2_ index and the incidence of POD was carried out through the application of either the independent samples t-test or the nonparametric rank-sum test. To ascertain the threshold value of the adjusted rSO_2_ index for predictive and evaluative purposes regarding POD in the pediatric population, the Receiver Operating Characteristics (ROC) curve was employed.

**Results:**

A total of 211 cases were included in this study, of which 61 (28.9%) developed POD. Participants suffering delirium had lower preoperative rSO_2_mean, lower preoperative rSO_2min_, and lower postoperative rSO_2min_, higher ∆rSO_2mean_, higher amount of ∆rSO_2mean_, lower ∆rSO_2min_ (*P* < 0.05). Preoperative rSO_2mean_ (AUC = 0.716, 95%CI 0.642–0.790), ∆rSO_2mean_ (AUC = 0.694, 95%CI 0.614–0.774), amount of ∆rSO_2mean_ (AUC = 0.649, 95%CI 0.564–0.734), preoperative rSO_2min_ (AUC = 0.702, 96%CI 0.628–0.777), postoperative rSO_2min_ (AUC = 0.717, 95%CI 0.647–0.787), and ∆rSO_2min_ (AUC = 0.714, 95%CI 0.638–0.790) performed well in sensitivity and specificity, and the best threshold were 62.05%, 1.27%, 2.41%, 55.68%, 57.36%, 1.29%.

**Conclusions:**

There is a close relationship between pediatric POD and rSO_2_. rSO_2_ could be used as an effective predictor of pediatric POD. It might be helpful to measure rSO_2_ with NIRS for early recognizing POD and making it possible for early intervention.

**Supplementary Information:**

The online version contains supplementary material available at 10.1186/s12888-024-05832-x.

## Key points

1. Pediatric postoperative delirium is closely related to rSO2.

2. Incorporating rSO2 monitoring into pediatric perioperative nursing plans and procedures could be effective, especially in younger children.

3. Further investigation can focus on different approaches to optimize perioperative cerebral oxygenation.

4. This study provides a new idea for the prediction, identification and evaluation of postoperative delirium in children, and helps to improve the objectivity, accuracy and effectiveness of evaluation.

**Supplementary Information:**

The online version contains supplementary material available at 10.1186/s12888-024-05832-x.

## Introduction

Postoperative delirium (POD) in pediatric patients is a common, transient complication following general anesthesia, characterized by fluctuating states of confusion during the post-anesthetic recovery phase [1]. Studies indicate a high incidence, with rates up to 66%, highlighting its significance in pediatric care [2, 3]. The inflammatory response to surgery contributes to cognitive deterioration [4], underscoring the risk of adverse events such as falls and unplanned extubation, which can extend hospital stays and increase healthcare costs [5, 6].

Recent advances in objective assessment tools for POD include glucose metabolism evaluation via positron emission tomography (PET) [7], neuroimaging techniques [8], and electroencephalography (EEG) [9]. Despite their potential, the implementation of such tools, especially EEG, in clinical settings remains inconsistent, reflecting the need for more reliable indicators tailored for pediatric patients.

In this context, Near-Infrared Spectroscopy (NIRS) has emerged as a crucial tool in this context, offering non-invasive monitoring of perioperative cerebral oxygenation [10, 11]. By analyzing the interaction of light with cerebral hemoglobins, NIRS provides real-time insights into brain oxygenation status, potentially linking cerebral tissue hypoperfusion and hypoxia to POD [12]. Recent studies have initiated investigations into the relationship between rSO_2_ and POD, indicating that perioperative rSO_2_ monitoring holds promise in the prediction, assessment, and identification of POD [11, 13]. Nevertheless, it is noteworthy that existing research is primarily focused on adult patients, with POD being more prevalent among elderly patients and those undergoing cardiac surgery, among other factors [11]. The specific relationship between perioperative rSO_2_ and POD in children following general surgery remains an area requiring thorough exploration.

Our study seeks to fill this gap by investigating rSO_2_’s potential as a predictive and diagnostic marker for POD in pediatric patients undergoing general anesthesia. We hypothesize that rSO_2_ can serve as a reflective indicator of POD, potentially improving the anticipation and management of this condition. By providing healthcare professionals with a reliable tool for anticipating and managing POD, we aim to enhance patient safety and care outcomes.

## Methods

### Study design and population

This study was carried out as a prospective observational study, focusing on pediatric patients who were hospitalized and in need of surgical treatment. The study cohort was recruited from Children’s Hospital, Zhejiang University School of Medicine, during the period spanning from November 2020 to March 2021. Exclusion criteria were applied to individuals meeting any of the following conditions: (a) the presence of factors that could potentially affect the assessment of delirium, such as severe cognitive impairment, coma, or deep sedation, (b) significant visual or hearing impairments that hindered the assessment of delirium, (c) participation in concurrent research endeavors involving new drugs or treatments, and (d) age falling below 1 year or exceeding 16 years. This study received approval from the Ethics Committee of Children’s Hospital, Zhejiang University School of Medicine on January 23, 2020, with the reference number 2020-IRB-001. In adherence to ethical standards, written informed consent was obtained from the parents of all participating children. Additionally, children who were 8 years of age or older provided their informed consent through a form specially designed for their age group.

### Study endpoints and power calculation

The endpoint for this study was the occurrence of postoperative delirium. The primary outcome measures were the predictive values of rSO_2_ values for delirium following surgery in pediatric patients. A sample size calculation was performed under the assumption that rSO_2_ could predict or identify the occurrence of postoperative delirium. Based on a previous study, the expected sensitivity and specificity were set at 91.67% and 79.31% respectively [14]. If the tolerance was set at 0.08, significance level at 0.05, according to the equation below, we needed 145 patients. Considering a 15% of follow-up loss, 167 patients were enrolled.

Sample size (n) based on sensitivity:

$${\text{n}}={\left(\frac{{\mu }_{\alpha }}{\delta }\right)}^{2}\left(1-{p}_{1}\right){p}_{1}$$

Sample size (n) based on specificity:

$${\text{n}}={\left(\frac{{\mu }_{\alpha }}{\delta }\right)}^{2}\left(1-{p}_{2}\right){p}_{2}$$

*p*1 = estimated sensitivity, *p*2 = estimated specificity, *μ*α = the value of *μ* in the normal distribution when the cumulative probability is equal to *α*/2, *δ* = tolerance (the value is generally 0.1 or 0.08).

### Anesthesia and postoperative pain management

For the administration of anesthesia and the management of postoperative pain, this study adhered to the established Standard Operating Procedures (SOPs) of Children’s Hospital, Zhejiang University School of Medicine, which are detailed in the Supplementary File. These SOPs are standardized to ensure consistency and ethical management of pediatric anesthesia and pain across different surgical procedures.

### Diagnosis of POD

The assessment of POD commenced immediately after the children regained consciousness following surgery. Evaluations were conducted every half hour over a 2-h period by a qualified psychiatrist. The DSM-5, considered the gold standard for identifying delirium, outlines several criteria for the diagnosis, including disturbances in attention, awareness, and cognition. These disturbances are not attributable to preexisting, established, or evolving neurocognitive disorders and represent a change from baseline attention and awareness. To diagnose POD, the psychiatrist specifically looked for acute onset and fluctuating levels of these disturbances, as observed through clinical assessment during the recovery phase. This method ensures sensitivity to the dynamic nature of delirium, where symptoms may come and go or increase in intensity throughout the observation period. Evaluations focused on the ability to direct, focus, sustain, and shift attention, and on the overall level of consciousness, which might range from hyperalertness to lethargy or stupor. To ensure the accuracy of the assessments, the psychiatrist remained in close proximity to the child throughout the evaluation period, minimizing the risk of overlooking any instances of delirium. This proximity allowed for immediate response and adjustment of the clinical assessment based on the child’s moment-to-moment changes in cognitive and perceptual disturbances. The psychiatrist’s assessments were detailed and recorded systematically to ensure that any occurrence of POD was captured accurately, providing a robust dataset for analysis and future reference.

### Monitoring of rSO_2_

The rSO_2_ was monitored using the NIRS (EGOS-600A, Aiqin, Suzhou, China). The rSO_2_ probes were placed on each children’s forehead and stabilized (single NIRS monitoring). Cerebral oxygen data were recorded every 2 s. We conducted rSO_2_ monitoring the day before surgery and after surgery as soon as the children awakened. Each monitoring event required 2 h. rSO_2_ (%) was calculated as follows: preoperative rSO_2_ (the average value of preoperative rSO_2_ detection values within 2 h); postoperative rSO_2_ (the average value of postoperative rSO_2_ detection values within 2 h); ∆rSO_2_ (%) = postoperative rSO_2_–preoperative rSO_2_; preoperative rSO_2min_ (the minimum preoperative rSO_2_ in 2 h); postoperative rSO_2min_ (the minimum postoperative rSO_2_ in 2 h); ∆rSO_2min_ (%) = postoperative rSO_2min_–preoperative rSO_2min_.

### Data collection

The data collection encompassed a wide range of information, including (a) general demographics: age, gender, weight, height, body mass index (BMI), and BMI Z-score; (b) Past medical history, which included prior surgical procedures, history of trauma, allergies, and the presence of major medical conditions, including but not limited to tic disorders; (c) Surgical-specific details, such as ASA (American Society of Anesthesiologists) classification, the duration of preoperative fasting and water deprivation, specifics regarding the anesthesia and surgical procedures, the volume of fluids administered intraoperatively, medication use, intraoperative bleeding, intraoperative body temperature monitoring, postoperative pain assessment, administration of oxygen, and the placement of drainage tubes.

### Statistical analysis

Means and standard deviations were used to summarized normally-distributed data, and medians and quartile ranges were used to summarize data with non-normal distributions. Univariate analyses (two-sample t test, Mann–Whitney U test, Pearson’s correlation, Spearman’s correlation) were performed to explore potential predictors and the correlation between rSO_2_ and POD. Variables related to POD (at *p* < 0.05) were used as predictors in multivariable logistic regression models. Variables related to rSO_2_ (at *p* < 0.05) were used as predictors in multivariable linear regression models and produced adjusted indicators of rSO_2_ (rSO_2_ indicators generated after correcting for confounders). The best cutoff values for the rSO_2_ on POD were further determined by receiver operating characteristic (ROC) analysis. Statistical significance was assessed at the 5% level (*p* < 0.05 was assumed to be statistically significant).

## Results

### General characteristics

**Fig. 1:**
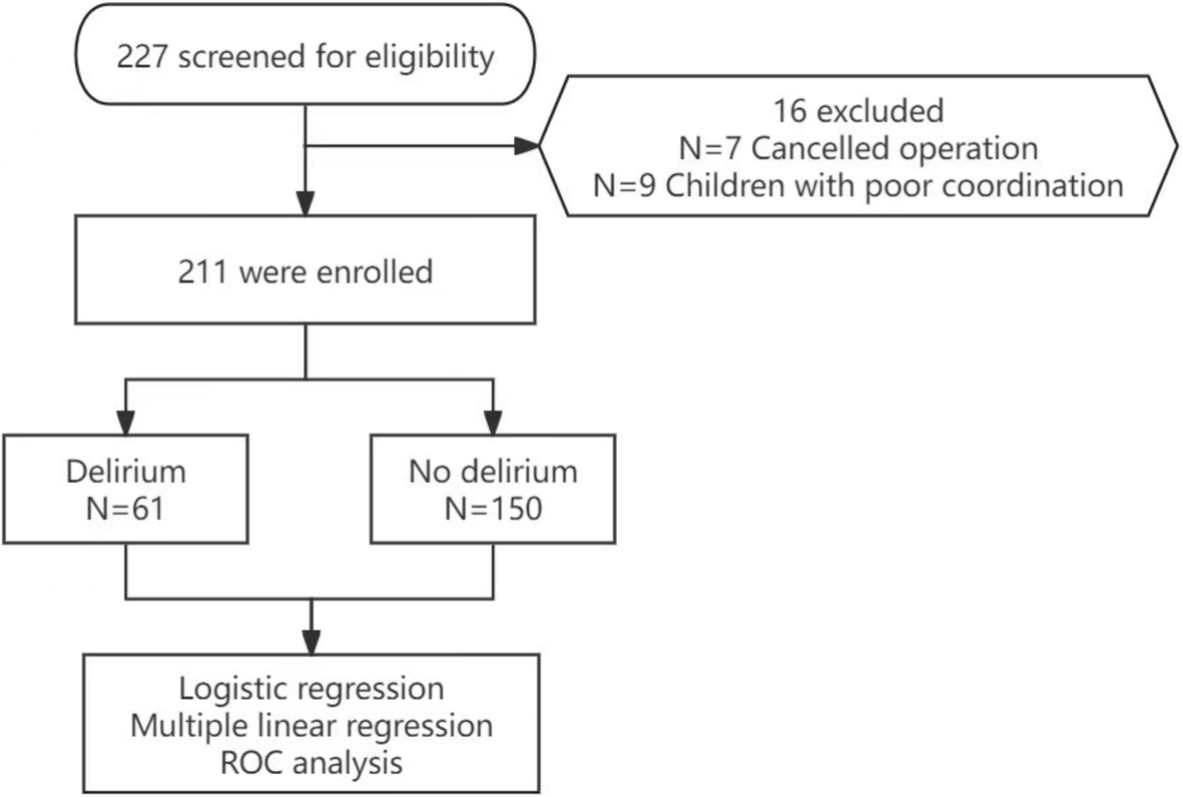
Flow chart for patient selection

A cohort of 211 pediatric participants were enrolled in the present study (Fig. 1). The median age of the cohort stood at 5 years, with a notable gender distribution, comprising 59.2% males. The majority of the pediatric subjects exhibited a Class I ASA physical status, with a prevalence of 85.8%. General anesthesia was administered to a substantial proportion of participants, employing tracheal intubation in 83.9% of cases. Noteworthy pharmaceutical agents employed during the surgical interventions included propofol and midazolam, each administered to all participants, as indicated in Table 1. Importantly, no rescue interventions were necessitated during the course of the surgical procedures. The logistic regression analysis showed that age, postoperative pain, and postoperative oxygen therapy could explain 31.5% of the variance in postoperative delirium among pediatric patients (Table 2).

**Table 1:** Demographic and clinical characteristics of the study participants (*N* = 211)

Variables	*n* (%)	Mean (*SD*) or Median (*P*_25_, *P*_75_)	Range
Gender			
Male	125 (59.2)		
Female	86 (40.8)		
Age (years)		5.00 (4.00, 7.00)	1.00–14.00
BMI Z-score^a^		-0.09 (1.42)	-4.48–5.91
History of operation and trauma	51 (24.2)		
History of allergy	32 (15.2)		
History of major diseases	8 (3.8)		
History of tic	4 (1.9)		
Duration of preoperative fasting (min)		764.77 (251.29)	127.00–1314.00
Duration of preoperative water deprivation (min)		401.00 (262.00, 664.00)	67.00–1314.00
ASA class^b^			
I	181 (85.8)		
II	30 (14.2)		
Anesthesia			
General anesthesia with trachea intubation	177 (83.9)		
General anesthesia without trachea intubation	34 (16.1)		
Surgical sites			
Ear-nose-throat	95 (45.0)		
Neck	13 (6.2)		
Limbs	42 (19.9)		
Abdomen	49 (23.2)		
Epidermal mass resection	12 (5.7)		
Operation			
With pneumoperitoneum	41 (19.4)		
Without pneumoperitoneum	170 (80.6)		
Medication			
Dexmedetomidine	122 (57.8)		
Glucocorticoids	174 (82.5)		
Antiemetics	170 (80.6)		
Anticholinesterase agents	41 (19.4)		
Muscarinic cholinoceptor blocking drugs	116 (55.0)		
Competitive muscular relaxants	164 (77.7)		
Inhalational anesthetics	34 (16.1)		
Opioid receptor agonists	174 (82.5)		
Opioid receptor partial agonists	63 (29.9)		
Benzodiazepines	100 (100.0)		
Duration of operation (min)		48.00 (38.00, 65.00)	20.00–410.00
Duration of anesthesia (min)		61.00 (52.00, 81.00)	29.00–440.00
Intraoperative liquid intake (ml)		150.00 (100.00, 200.00)	80.00–2300.00
Intraoperative bleeding (ml)		1.00 (1.00, 2.00)	1.00–80.00
Intraoperative body temperature (℃)		36.80 (36.50, 37.00)	36.00–37.80
Postoperative pain	160 (75.8)		
Postoperative receiving oxygen	90 (42.7)		
Drainage tube	15 (7.1)		

^a^*ASA* American Society of Anesthesiologists

^b^*BMI* body mass index

**Table 2:** Logistic regression for postoperative delirium (*N* = 211)

Variables	*Β*	*SE*	Wald	*p* Value	95% *CI*
Age (years)	0.259	0.079	10.709	0.001^**^	1.109–1.512
Postoperative pain	2.154	0.576	13.984	< 0.001^**^	2.787–26.649
Postoperative receiving oxygen	1.331	0.356	14.025	< 0.001^**^	1.886–7.600

*n/a* not applicable

*χ*^2^ = 52.569

^**^p < 0.01

### Preoperative rSO_2_, postoperative rSO_2_ and ∆rSO_2_

The preoperative rSO_2_ was quantified at (62.19 ± 2.55)%, with a median preoperative rSO_2min_ of 56.35% (53.22–58.48). Following the surgical procedure, postoperative rSO_2_ levels were recorded as (64.02 ± 3.18)%, and the median postoperative rSO_2min_ was 58.09 (53.99–60.61)%. The ∆rSO_2_ was (1.83 ± 3.35)%, with the median value of ∆rSO_2_ amounting to 2.42% (1.00–4.49), and ∆rSO_2min_ was (1.19 ± 5.98)%. Tables 3 and 4 present the predictors associated with preoperative rSO_2_, postoperative rSO_2_, and ∆rSO_2_. In order to control for potential confounding factors, adjusted values for these three indicators were computed, taking into account variables such as age, the use of antiemetics, administration of dexmedetomidine, postoperative pain levels, and the provision of oxygen after surgery.

**Table 3:** Multiple linear regression for rSO_2_ (*N* = 211)

Variables	Preoperative rSO_2_		Postoperative rSO_2_		∆rSO_2_	
	*B*	*p* value	*B*	*p* value	*B*	*p* value
(Constant)	60.737	< 0.001^**^	68.257	< 0.001^**^	7.557	< 0.001^**^
Age	0.264	< 0.001^**^	0.290	< 0.001^**^	n/a	n/a
Antiemetics	n/a	n/a	-1.910	< 0.001^**^	-1.877	0.001^**^
Postoperative receiving oxygen	n/a	n/a	-2.259	< 0.001^**^	-2.216	< 0.001^**^

Multiple linear regression for preoperative rSO_2_: *R*^2^ = 0.085, *F* = 19.514, *p* < 0.001

Multiple linear regression for postoperative rSO_2_: *R*^2^ = 0.231, *F* = 20.766, *p* < 0.001

Multiple linear regression for ∆rSO_2_: *R*^2^ = 0.194, *F* = 25.065, *p* < 0.001

*n/a* not applicable

^**^p < 0.01

**Table 4:** Multiple linear regression for rSO_2_min (*N* = 211)

Variables	Preoperative rSO_2_min		Postoperative rSO_2_min		∆rSO_2_min	
	*B*	*p* value	*B*	*p* value	*B*	*p* value
(Constant)	54.299	< 0.001^**^	48.137	< 0.001^**^	-6.182	0.001^**^
Age	0.282	0.007^**^	n/a	n/a	n/a	n/a
Dexmedetomidine	n/a	n/a	1.993	0.005^**^	n/a	n/a
Postoperative pain	n/a	n/a	2.901	< 0.001^**^	3.023	0.001^**^
Postoperative receiving oxygen	n/a	n/a	1.569	0.028^*^	2.297	0.005^**^

Multiple linear regression for preoperative rSO_2_min: *R*^2^ = 0.034, *F* = 7.454, *p* = 0.007

Multiple linear regression for postoperative rSO_2_min: *R*^2^ = 0.115, *F* = 8.947, *p* < 0.001

Multiple linear regression for ∆rSO_2_min: *R*^2^ = 0.074, *F* = 8.258, *p* < 0.001

*n/a* not applicable

^*^p < 0.05

^**^p < 0.01

### Correlation between POD and rSO_2_

In the context of POD among children, all rSO_2_ values demonstrated significant associations, with the exception of postoperative rSO_2_ adjustments, which did not exhibit a statistically significant relationship with the occurrence of POD. Specifically, participants who experienced delirium following surgery displayed several noteworthy trends in their adjusted rSO_2_ values. These included lower adjusted preoperative rSO_2_ levels (z = -4.992, *p* < 0.001), decreased adjusted preoperative rSO_2min_ (z = -4.606, *p* < 0.001), reduced adjusted postoperative rSO_2min_ (z = -4.942, *p* < 0.001), elevated adjusted ∆rSO_2_ (z = -4.416, *p* < 0.001), and diminished adjusted ∆rSO_2min_ (z = -4.865, *p* < 0.001), as detailed in Table 5.

**Table 5:** The correlations between rSO_2_ and postoperative delirium (*N* = 211)

Variables	Delirium (*N* = 61)	No Delirium (*N* = 150)	*t* / *z*	*p* value
Adj-preoperative rSO_2_ (%)	61.79 (61.53, 62.04)	62.06 (61.79, 62.84)	-4.922	< 0.001^**^
Adj-postoperative rSO_2_ (%)	64.97 (63.04, 65.25)	63.60 (62.90, 65.25)	-1.716	0.086
Adj-∆rSO_2_ (%)	3.44 (1.27, 3.48)	1.26 (1.21, 3.44)	-4.416	< 0.001^**^
Adj-preoperative rSO_2_min (%)	55.41 (55.14, 55.69)	55.72 (55.41, 56.52)	-4.606	< 0.001^**^
Adj-postoperative rSO_2_min (%)	56.19 (54.66, 56.63)	58.06 (56.14, 58.26)	-4.942	< 0.001^**^
Adj-∆rSO_2_min (%)	-0.73 (-0.86, 1.45)	1.45 (1.38, 2.13)	-4.865	< 0.001^**^

*Adj* adjusted

^**^p < 0.01

### The ROC analysis for rSO_2_ on POD

**Fig. 2:**
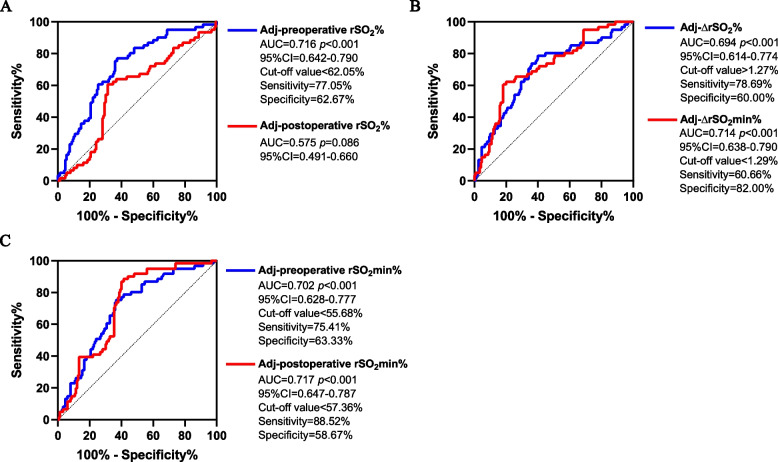
The ROC curves for rSO_2_ on postoperative delirium

Results of ROC analysis for rSO_2_ on POD are shown in Fig. 2. Adjusted preoperative rSO_2_ (AUC = 0.716, 95%CI 0.642–0.790, *p* < 0.001), ∆rSO_2_ (AUC = 0.694, 95%CI 0.614–0.774, *p* < 0.001), preoperative rSO_2min_ (AUC = 0.70_2_, 96%CI 0.628–0.777, *p* < 0.001), postoperative rSO_2min_ (AUC = 0.717, 95%CI 0.647–0.787, *p* < 0.001), and ∆rSO_2min_ (AUC = 0.714, 95%CI 0.638–0.790, *p* < 0.001) performed well in sensitivity and specificity.

## Discussion

In our current study, we conducted a comprehensive follow-up of 211 pediatric surgery patients and identified that 28.9% of them developed POD, as diagnosed in accordance with the DSM-5 criteria. Based on our initial hypothesis suggesting a close association between regional cerebral oxygen saturation (rSO2) and the occurrence of POD, we systematically monitored both preoperative and postoperative rSO2 levels in our cohort of patients. Through our investigation, we successfully validated the strong relationship between preoperative and postoperative rSO2 and the development of POD. Furthermore, our study revealed predictive values of rSO2 that can serve as valuable indicators for assessing the likelihood of POD.

Utilizing NIRS to assess rSO_2_, our study observed that the mean rSO_2_ values in our participant cohort fell within the normal range (preoperative rSO_2_ = 62.19, SD = 2.55; postoperative rSO_2_ = 64.02, SD = 3.18), as stipulated within the reported range of 60%-70% [15, 16]. Furthermore, our investigation identified age as the most influential predictor of both preoperative and postoperative rSO_2_ levels. Specifically, older children displayed higher preoperative rSO_2_, increased preoperative rSO_2min_, and elevated postoperative rSO_2_ values. This phenomenon can be attributed to the rapid developmental changes occurring in the pediatric brain, leading to heightened cerebral blood flow compared to adults [17]. Interestingly, this finding aligns with a prior study involving children aged 7–13 years, which also reported age as a positive predictor of cerebral oxygenation [18]. However, it contrasts with some studies in adults that have shown a negative correlation between age and rSO_2_ [19]. This discrepancy may be attributed to the fundamental differences in brain physiology between adults and children, which results in distinct age-related patterns of rSO_2_, emphasizing the importance of considering age as a significant factor in pediatric studies. In addition to age, our study identified postoperative pain, administration of postoperative oxygen, and the utilization of specific medications as significant predictive factors for postoperative rSO_2_. Notably, the provision of postoperative oxygen exhibited a positive effect, leading to an increase in both postoperative rSO_2_ and ∆rSO_2_ levels. This observation suggests that the postoperative rSO_2_ tends to surpass the preoperative rSO_2_, which may be attributed to intraoperative ventilation practices or the administration of high oxygen concentrations. Moreover, our study revealed that postoperative rSO_2min_ and ∆rSO_2min_ values were notably lower in children who received oxygenation following surgery. This implies that the actual postoperative rSO_2_ levels in the oxygenated group were inferior to those in the non-oxygenated group. The decrease in postoperative rSO_2min_ and ∆rSO_2min_ associated with postoperative pain aligns with findings from previous studies, likely attributable to the established link between pain and reduced cerebral blood flow [20, 21].

Certain medications have been found to influence postoperative rSO_2_, including dexmedetomidine and antiemetics. Dexmedetomidine is known to effectively reduce postoperative agitation in pediatric patients undergoing general anesthesia, which is why it is commonly used as a preventive measure against POD [22]. However, our study revealed that children who received dexmedetomidine exhibited lower postoperative rSO_2min_. One possible explanation for this observation is that dexmedetomidine, which can pass through the blood–brain barrier, exerts a central anti-sympathetic effect, inhibiting the release of catecholamines, thereby reducing blood pressure and slowing heart rate [23]. In our research, the use of antiemetics (ondansetron) was associated with higher postoperative rSO_2_ and ∆rSO_2_. This association may be attributed to the intravenous administration of ondansetron, which helps maintain hemodynamic stability. This, in turn, can reduce the incidence of post-anesthetic hypotension, bradycardia, and tremors.

Given the intricate interplay of confounding factors affecting rSO_2_, our study employed multiple linear regression to calculate adjusted rSO_2_ indicators. Subsequently, we conducted an analysis to explore the relationship between rSO_2_ and the occurrence of POD. Our investigation identified five rSO_2_ indicators that demonstrated strong predictive capability for POD, encompassing both adjusted and unadjusted parameters. Primarily, our study unveiled a substantial influence of preoperative rSO_2_ on the likelihood of POD, particularly highlighting the significance of smaller values in the context of adjusted preoperative rSO_2_ and adjusted preoperative rSO_2min_. This observation underscores the critical importance of preoperative rSO_2_ measurements. It’s worth noting that various researchers have proposed the concept of cognitive reserve, and building upon this idea, Julika and colleagues [24] have suggested that rSO_2_ could be viewed as a physical marker of cognitive reserve. In the context of our study, the lower preoperative rSO_2_ levels observed in children who subsequently developed POD may be indicative of heightened susceptibility to cerebral impairment. When compared to prior findings in adult populations, our study revealed a superior predictive value of adjusted preoperative rSO_2_, with a threshold of less than 62.05%. This value is notably higher than the reported threshold of less than 59.5% in adults [25]. This discrepancy may be attributed to the fundamental physiological distinctions between adults and children. Collectively, our findings underscore the critical importance of monitoring preoperative rSO_2_ in pediatric surgical cases. Specifically, if the preoperative mean rSO_2_ falls below 62.05% or the minimum rSO_2_ is less than 55.68%, heightened vigilance for the potential development of POD is warranted.

Furthermore, our analysis revealed that diminished values of the adjusted postoperative rSO_2min_ were predictive of POD, with a threshold set at 57.36%. Although establishing a causal relationship between low postoperative rSO_2_ and the subsequent occurrence of POD posed challenges, the clear correlation between decreased postoperative rSO_2min_ and the presence of POD underscores the critical need for healthcare providers to exercise enhanced vigilance when attending to pediatric patients displaying lower postoperative rSO_2min_ values. In addition, our investigation indicated that elevated values of the adjusted ∆rSO_2_ and reduced values of the adjusted ∆rSO_2min_ were associated with an increased likelihood of POD. In our study, adjusted ∆rSO_2_ values exceeding 1.27% and adjusted ∆rSO_2min_ values below 1.29% were indicative of a heightened risk of POD. It is essential to acknowledge that ∆rSO_2_ values can be influenced by subtle factors such as sensor positioning, scattering, and variations in the path length of the detected light beam. Consequently, further research is warranted to confirm the association between ∆rSO_2_ and POD.

### Implications for clinical practice

This study reveals the potential of rSO2 as an indicator for postoperative delirium (POD) in children, suggesting that perioperative monitoring of cerebral oxygen saturation could be crucial for early detection and intervention. The implementation of rSO2 monitoring could enable healthcare providers to identify patients at risk of POD, potentially leading to tailored care strategies that improve postoperative recovery. Future research should aim to define rSO2 thresholds for intervention and evaluate the effectiveness of such measures in reducing POD. Our findings advocate for a paradigm shift in perioperative care, emphasizing cerebral oxygenation as a key factor in pediatric anesthesia management.

### Limitations

Several limitations should be considered in the context of this study. Firstly, the exclusive recruitment of children from a single medical center may limit the generalizability of the findings. Secondly, it’s important to acknowledge that the predictors examined in this study only accounted for a portion of the variance in rSO2. To obtain a more comprehensive understanding, further investigations are in the planning stages to address this issue in greater detail. Thirdly, our study did not delve into the prediction of postoperative delirium by rSO2 within specific age groups. Subsequent research should aim to establish rSO2 thresholds for predicting postoperative delirium in children of varying age brackets. Moreover, it is essential to recognize that confounding variables were not entirely controlled for, owing to certain clinical constraints. Additionally, the study was hindered by insufficient intraoperative monitoring of the children’s condition. We also acknowledge the absence of comprehensive electrolyte monitoring as a significant limitation, which could have provided additional insights into the perioperative physiological changes affecting our patients. Finally, it’s important to note that delirium episodes occurring more than 2 h after surgery were not considered in this study, as the pediatric patients generally exhibited mild conditions and rapid postoperative recovery. Additionally, the heterogeneity in anesthesia and analgesia protocols might have influenced the study outcomes related to POD. Although we meticulously incorporated various anesthesia-related factors into our analysis, the variability in these protocols could pose a challenge to the consistency of our results, which we plan to address more thoroughly in future research.

## Conclusion

In conclusion, our study underscores the potential of rSO_2_, as measured by NIRS, as a valuable predictor of pediatric POD. However, it is crucial to emphasize that further validation through large-scale, multi-center studies is essential to solidify this relationship. In terms of prevention and treatment, interventions should be tailored to optimize perioperative cerebral oxygenation through various approaches, including the optimization of oxygen content, hemoglobin levels, and hemodynamic status.

### Supplementary Information

Supplementary Material 1.

Supplementary Material 2.

## Data Availability

The datasets used during the current study available from the corresponding author on reasonable request.
