# Supplementary material for: Association between pediatric postoperative delirium and regional cerebral oxygen saturation: a prospective observational study
Source: BMC Psychiatry. 2024 May 15;24:367. doi: 10.1186/s12888-024-05832-x (PMC11097584; doi:10.1186/s12888-024-05832-x)
Supplement: Supplementary file 1 — Supplementary Material 1. [file 12888_2024_5832_MOESM1_ESM.docx]

**Supplementary Files**

**Standard Operating Procedure (SOP) for Pre-anesthesia Preparation and Anesthetic Management**

**I. Pre-anesthesia Preparation**

**1. Pre-anesthesia Evaluation and Assessment**

- Collect detailed history regarding the pediatric patient's medical condition, growth and development, physical examination findings, auxiliary examinations, and comorbidities.
- Establish trust and cooperation with the child and their parents to minimize anxiety.

1. **Fasting and Fluid Restrictions Before Anesthesia**

- Follow the standardized guidelines strictly. Treat emergency surgeries as if the stomach is full.

1. **Pre-anesthesia Medication**

- Administer midazolam syrup orally 10-20 minutes before anesthesia induction at a dose of 0.25-0.5mg/kg, with a maximum recommended dose of 10mg.
- For uncooperative children, add 3mg/kg ketamine to the syrup or administer phenobarbital sodium 2mg/kg intramuscularly 30 minutes before anesthesia. Administer anticholinergic drug, glycopyrrolate, at a dose of 0.01mg/kg during anesthesia induction.

1. **Venous Access**

- After entering the operating room, use a 20-24G cannula to ensure smooth infusion.
- Administer ketamine 5mg/kg intramuscularly to uncooperative children before anesthesia induction and room entry.

1. **Anesthesia Equipment Check**

- Perform rigorous checks of the anesthesia machine, monitoring devices, and related equipment.
- Prepare specialized pediatric intubation and ventilation tools such as pediatric tubes, circuits, reservoir bags, masks, oropharyngeal airways, and small laryngoscopes. Prepare puncture tools for spinal anesthesia.

1. **Anesthesia Monitoring**

- Basic monitoring includes non-invasive blood pressure, electrocardiogram, and pulse oximetry; add end-tidal CO2 monitoring for general anesthesia.
- Enhance monitoring with invasive arterial and venous pressure measurements in cases of significant hemodynamic fluctuations or substantial blood loss. Monitor arterial blood gases during prolonged mechanical ventilation and adjust ventilation parameters based on gas analysis.

1. **Medication Preparation**

- Prepare anesthetic drugs, vasoactive medications, and other necessary pharmaceuticals as planned.
- Dilute medications as required, e.g., midazolam 1mg/ml, sufentanil 2.5μg/ml, remifentanil 25μg/ml, propofol 10mg/ml, vecuronium 1mg/ml, ephedrine 6mg/ml, atropine 0.1mg/ml.

**II. Anesthetic Implementation and Management**

**1. Basic Anesthesia**

- Administer ketamine 5mg/kg intramuscularly for uncooperative children or those unable to enter the operating room.
- Note: Reduce the dosage in cases of preoperative partial airway obstruction, respiratory depression, severe anemia, or inadequate blood volume.

1. **Mask Inhalation Anesthesia**

- Indications include surgeries with no risk of regurgitation and aspiration, difficult airways, and emergency resuscitation.
- Contraindications include full stomach, high intra-abdominal pressure with a high risk of regurgitation and aspiration, facial deformities, pathological changes in the oropharynx, excessive secretions, laryngeal infection or other pathological changes, respiratory tract bleeding, and surgeries in the oropharyngeal area.
- Methods include selecting an appropriate mask that fits the child's face and minimizes dead space, supporting the jaw to keep the airway open, and using fingers to seal the mask to reduce air leaks.

1. **Epidural Anesthesia for Children**

- Indications mainly include lower abdominal, pelvic, or lower limb surgeries.
- Contraindications include infection at the puncture site and coagulation disorders.
- Techniques involve positioning the child, ensuring asepsis, advancing the needle with saline or air in the syringe until loss of resistance, and administering local anesthetics once epidural space is confirmed.

1. **Caudal Block**

- Indications include surgeries on the perineum, lower limbs, or groin.
- Contraindications include infection or skin abnormalities at the puncture site and coagulation disorders.
- Techniques include performing under sedation or basic anesthesia, positioning the child, locating the sacral hiatus, disinfecting the area, and using a caudal needle to inject local anesthetics once the sacral canal is accessed.

1. **Tracheal Intubation/Supraglottic Airway Anesthesia**

- Indications: Ensure airway patency, prevent regurgitation and aspiration, required for prolonged positive pressure ventilation, and for repetitive suctioning of tracheal secretions.
- Contraindications: Acute upper respiratory or pulmonary infections.
- Supraglottic airway is indicated in surgeries without risk of vomiting and aspiration, especially in difficult intubation scenarios, and can be used as a guide for tracheal intubation.

1. **Intravenous Anesthetic Induction and Maintenance**

- Use medications like midazolam, fentanyl or sufentanil, and propofol at dosages adjusted based on the clinical situation and requirements.
- Maintain anesthesia primarily with IV agents like propofol, supplemented with inhaled anesthetics as needed, and adjust analgesics and muscle relaxants accordingly.

1. **Respiratory Management and Recovery from Anesthesia**

- Set appropriate ventilation parameters including tidal volume, inspiratory to expiratory ratio, and inspiratory pressure.
- Criteria for extubation include recovery from anesthesia effects, spontaneous breathing, and stable circulatory function without hypothermia.

1. **Fluid Management Principles**

- Calculate daily and hourly fluid requirements based on weight.
- Estimate blood volume, predict and measure blood loss, and adjust fluid therapy accordingly.
- Include preoperative fasting volume, intraoperative losses, and physiological maintenance in fluid therapy plans.

**Standard Operating Procedure (SOP) for Pediatric Pain Assessment and Management**

**I. Pediatric Pain Assessment**

**1. Self-assessment Methods**

- Visual Analog Scale (VAS): Suitable for children aged 8 years and above. It involves a 100mm line ranging from "no pain" to "worst imaginable pain".
- Numerical Rating Scale (NRS): Applicable for children aged 8 years and above. It uses a scale from 0 to 10, where "0" indicates no pain and "10" signifies extreme pain.

1. **Behavioral Assessment**

- CRIES Scale: Designed for non-verbal infants, this scale evaluates crying, requirement for oxygen supplementation, vital signs, facial expressions, and sleep patterns.
- FLACC Scale: Used for children aged 1 to 18 years, assessing five parameters: Face, Legs, Activity, Cry, and Consolability.
- CHEOPS Scale: Applicable for children older than 6 months, focusing on crying, facial expression, verbal response, activity of legs, torso movement, and touch sensitivity around the wound.

1. **Physiological Assessment**

- Integrates physiological signs such as heart rate, respiration, and blood pressure, usually combined with other assessment tools for comprehensive evaluation.

**II. Principles of Postoperative Pain Management in Pediatrics**

**1. Multimodal Pain Management**

- Pharmacological Treatment: Includes acetaminophen, nonsteroidal anti-inflammatory drugs (NSAIDs), tramadol, codeine, and potent opioids.
- Technical Approaches: Techniques such as regional blocks, epidural analgesia, intravenous pain management, and local infiltration.
- Non-pharmacological Methods: Methods such as pacifiers, sucrose, massage, and music therapy are employed to alleviate pain.

1. **Patient-Controlled Analgesia (PCA) and Nurse-Controlled Analgesia (NCA):**

- Allows patients or caregivers to manage pain medication through a controlled infusion pump, offering personalized dose adjustment based on real-time needs.

**III. Implementation and Monitoring**

**1. Assessment of Pain Relief:** It is essential to evaluate the effectiveness of pain relief measures and identify any adverse reactions post-treatment.

**2. Monitoring and Adjustments:** Adjustments to the pain management plan should be made based on ongoing pain assessments and patient feedback.

**3. Education and Training:** Healthcare providers should receive training on pain management techniques to enhance their expertise and accuracy in assessing and managing pain.
