# Supplementary material for: Association between pediatric postoperative delirium and regional cerebral oxygen saturation: a prospective observational study
Source: BMC Psychiatry. 2024 May 15;24:367. doi: 10.1186/s12888-024-05832-x (PMC11097584; doi:10.1186/s12888-024-05832-x)
Supplement: Supplementary file 2 — Supplementary Material 2. [file 12888_2024_5832_MOESM2_ESM.docx]

**Supplementary Table 1.** Univariate Analysis of Postoperative Delirium (*N*=211)

| Variables | Delirium (*N*=61) | No-Delirium (*N*=150) | *z*/*χ*^2^ | *p* Value |
| --- | --- | --- | --- | --- |
| Age (years) | 4.00 (3.00, 5.00) | 5.00 (4.00, 8.00) | -4.858 | <0.001** |
| History of allergy |  |  | 5.924 | 0.015* |
| Yes | 15 (24.59) | 17 (11.33) |  |  |
| Surgical sites |  |  | 9.784 | 0.044* |
| Ear-nose-throat | 35 (57.38) | 60 (40.00) |  |  |
| Neck | 4 (6.56) | 9 (6.00) |  |  |
| Limbs | 5 (8.20) | 37 (24.67) |  |  |
| Abdomen | 15 (24.59) | 34 (22.67) |  |  |
| Epidermal mass resection | 2 (3.28) | 10 (6.67) |  |  |
| Anesthesia |  |  | 3.979 | 0.046* |
| General anesthesia with trachea intubation | 56 (91.80) | 121 (80.67) |  |  |
| General anesthesia without trachea intubation | 5 (8.20) | 29 (19.33) |  |  |
| Dexmedetomidine |  |  | 7.206 | 0.007** |
| Yes | 44 (72.13) | 78 (52.00) |  |  |
| Competitive muscular relaxants |  |  | 5.780 | 0.016* |
| Yes | 54 (88.52) | 110 (73.33) |  |  |
| Opioid receptor agonists |  |  | 5.175 | 0.023* |
| Yes | 56 (91.80) | 118 (78.67) |  |  |
| Opioid receptor partial agonists |  |  | 5.729 | 0.017* |
| Yes | 11 (18.03) | 52 (34.67) |  |  |
| Postoperative pain |  |  | 14.524 | <0.001** |
| Yes | 57 (93.44) | 103 (68.67) |  |  |
| Postoperative receiving oxygen |  |  | 15.886 | <0.001** |
| Yes | 39 (63.93) | 51 (34.00) |  |  |

*Note*. **p*<0.05, ***p*<0.01.
